# Non-invasive electrical cardiometry cardiac output monitoring during prehospital helicopter emergency medical care: a feasibility study

**DOI:** 10.1007/s10877-021-00657-5

**Published:** 2021-01-23

**Authors:** Cornelis Slagt, Sjoerd Servaas, Rein Ketelaars, Geert-Jan van Geffen, Marijn Cornelia Theresia Tacken, Corien Alexandra Verrips, Lonneke Ankie Marcel Baggen, Gert Jan Scheffer, Lucas Theodorus van Eijk

**Affiliations:** 1 Department of Anesthesiology, Pain and Palliative Medicine, Radboud University Nijmegen Medical Centre, Geert Grooteplein Zuid 10, Huispost 717, route 714, Postbus 9101, 6500 HB Nijmegen, The Netherlands; 2 Helicopter Emergency Medical Service Lifeliner 3, Geert Grooteplein Zuid 10, 6500 HB Nijmegen, The Netherlands

**Keywords:** Non-invasive hemodynamic monitoring, Electrical cardiometry, Prehospital helicopter care, Cardiac output

## Abstract

**Purpose:**

Introducing advanced hemodynamic monitoring might be beneficial during Helicopter Emergency Medical Service (HEMS) care. However, it should not increase the on-scene-time, it should be easy to use and should be non-invasive. The goal of this study was to investigate the feasibility of non-invasive cardiac output measurements by electrical cardiometry (EC) and the quality of the EC signal during pre-hospital care provided by our HEMS.

**Methods:**

A convenience sample of fifty patients who required HEMS assistance were included in this study. Problems with respect to connecting the patient, entering patient characteristics and measuring were inventoried. Quality of EC signal of the measurements was assessed during prehospital helicopter care. We recorded the number of measurements with a signal quality indicator (SQI) ≥ 80 and the number of patients having at least 1 measurement with a SQI ≥ 80. Furthermore, the SQI value distribution of the measurements within each patient was analysed.

**Results:**

In the experience of the attending HEMS caregivers application of the device was easy and did not result in increased duration of on-scene time. Patch adhesion was reported as a concern due to clammy skin in 22% of all cases. 684 measurements were recorded during HEMS care. In 47 (94%) patients at least 1 measurement with an SQI ≥ 80 was registered. Of all recorded measurements 5.8% had an SQI < 40, 11.4% had an SQI 40–59, 14.9% had a SQI between 60 and 79 and 67.8% had SQI ≥ 80.

**Conclusion:**

Cardiac output measurements are feasible during prehospital HEMS care with good quality of the EC signal. Monitoring was easy to use and quick to install. In our view it is an promising candidate for the prehospital setting. Further research is needed to determine its clinical value during clinical decision making.

## Purpose

Death from trauma is the leading cause of death among people aged 5–30 years worldwide [1]. Most deaths occur in het prehospital setting from massive exsanguination or severe head trauma [2]. Main goals of prehospital care are optimal resuscitation and transportation to a suitable medical/surgical facility [3]. The identification and treatment of occult shock remains pivotal to prevent later deaths [4]. Shock is a state of hypoperfusion at the cellular level, when oxygen delivery is in disbalance with oxygen consumption. Optimization of cardiac output (CO) is key to the treatment of shock [4] and CO is a predictor for survival in trauma patients [5].

Nowadays patients in the prehospital setting are monitored using heart rate, electrocardiogram (ECG), peripheral saturation and non-invasive blood pressure. Advanced haemodynamic monitoring may be of benefit, as in high risk surgery patients it has been shown to positively influence morbidity, mortality and in-hospital length of stay when the right hemodynamic goals are achieved [6, 7]. However, the invasive measurement of cardiac output is considered undesirable in the prehospital period, as it takes valuable time to install, requires a sterile environment to insert and equipment is voluminous. Therefore, data on the use of advanced hemodynamic measurements in the prehospital care are lacking. Introducing new monitoring techniques may aid prehospital caregivers in clinical decision making. Such techniques should preferably not increase on-scene time and should be low in weight and volume, easy to use and be non-invasive [3, 5]. Recent studies show that non-invasive advanced haemodynamic measurement methods can be used in regular care as long as their limitations are known [8].

Thoracic electrical bioimpedance (TEB) was first described in 1966 by Kubicek et al. [9]. This method is based on changes in thoracic resistance as a result of changes in blood velocity during the cardiac cycle and uses an algorithm to calculate the CO. The algorithm has been modified over time. The most recent modification was performed by Bernstein and Osypka in 200, the called it electrical cardiometry (EC) [10, 11]. The ICON® hemodynamic measuring device (Osypka Medical GmbH, Germany) uses this latest algorithm modification and is a non-invasive hemodynamic measuring device. It is a robust and portable handheld device, and may be perfectly suited for the use in prehospital care. Recently it was used in the initial hemodynamic evaluation of trauma patients in the emergency department [12]. The device has its own internal quality score to assess the quality of the measured signal. This so called Signal Quality Indicator (SQI) should be at least 80 for the measurements to be used for clinical decision making [13]. EC has shown to be safe and easy to use, with accuracy and precision equal to other less invasive measuring devices [12, 14, 15].

The goal of this study was to investigate the feasibility of performing non-invasive measurements using the ICON® device during the pre-hospital care provided by the Helicopter Emergency Medical Service (HEMS), Lifeliner 3, the Netherlands. The feasibility was evaluated by assessing the quality of measurements by looking at the SQI, and by assessing user experience.

## Methods

An explorative feasibility study was performed during prehospital helicopter care in a convenience sample of 50 critically ill patients who required acute assistance of the HEMS. Patients were included from May 2017–June 2018. This study was approved by the medical ethical committee Arnhem-Nijmegen, the Netherlands, file 2017-3203. They waived the need for informed consent due to the non-invasive character of the study. The study was registered at www.trialregister.nl, NTR5249-NL7250.

### Cardiac output measurement by electrical cardiometry

Before the study was started, doctors and nurses were trained in the use of the ICON® monitor. After arrival of the HEMS on the scene, and after initial clinical evaluation and treatment, the EC device was connected to the patient as soon as the clinical condition would allow. This could be on the trauma scene, or during transport to the hospital via helicopter or regular ambulance. After turning on the device, it performs a 60 s self-test. In the meanwhile the skin sensors (total 4) were placed on the neck (either left or right side) and left side of the thorax according to the operational manual provided by the manufacturer. These allow the continuous measurement of the changes of electrical conductivity within the thorax in response to a low amplitude, high frequency electrical current. Filtering techniques isolate changes in conductivity created by the circulatory system, which is mainly determined by blood in the aorta and its change in conductivity when subjected to pulsatile blood flow before and after aortic valve opening. This is used to derive the peak aortic acceleration (ACC) and left ventricle ejection time (LVET). Stroke volume is calculated using patient characteristics (gender, age, length, body weight), ACC and LVET [11, 16]. Further details of the device are described elsewhere [16, 17].

After connecting the patient and entering the patient characteristics, continuous measurements were started. Measurements were started within 2 min after connection to the patient. If patient characteristics were unknown, these were estimated by the HEMS crew. As artefacts may lead to unreliable estimates of cardiac output, the monitor provides an internal quality indicator of the data signal expressed as SQI. An SQI of ≥ 80 is associated with high-quality advanced haemodynamic measurements as per manufacturer recommendations [13, 17, 18]. The EC device used for this study provides a SQI for each measurement, representing signal strength, adding to the reliability of the device. The SQI is based on two signal criteria. First, the signal has to meet pre-programmed shape and time requirements. Second, the signal magnitude has to be within certain statistical limits. An SQI of 80 means 8 out of 10 cardiac cycles were acceptable with respect to shape, time, magnitude and statistical limits [18]. SQI is displayed on the monitor in the top left corner as bars, each bar representing 20%. Data were stored per 1 min interval and were analysed later. HEMS care providers were instructed not to use the data for clinical decision making and that measurements were performed for research purposes only. The device was not blinded, so that care givers were able see the SQI indicator on the device and if necessary could check connections if SQI was/became low.

### Data quality assessment

The primary endpoint was quality of the data as reflected by the signal quality indicator SQI. As no data exists on the quality of ICON measurements in prehospital care, we recorded the number and proportion of measurements with a SQI ≥ 80 and the number of patients having at least 1 measurement with a SQI ≥ 80. Furthermore, the SQI value distribution of the measurements within each patient was analysed. Data were analysed using the appropriate tests in GraphPad Prism version 5.03 (GraphPad software, San Diego, USA). Data were assessed for normal distribution using the D’Agostino & Pearson omnibus normality test. Not normally distributed data were analysed using the Mann Whitney test. A P-value < 0.05 was considered statistically significant.

### User experience

The secondary endpoint was user experience. Users reported their experiences in the electronic medical patient registry. The quality and number of data per patient was related to the reported user experience to check whether any specific problems were associated with poor measurements.

### Feasibility evaluation

Feasibility was evaluated based on quality of the data, and user experience, where both had to be acceptable to qualify the EC measurements using the ICON device as feasible; no insurmountable user-reported problems had to be present in > 90% of the patients, and in > 90% of the patients at least 1 measurement with an SQI had to be ≥ 80.

### Setting

The Netherlands are covered by four HEMS teams, all affiliated with a level 1 trauma center. The general goal of the HEMS teams is to quickly deliver medical care provided by a medical specialist (anesthesiologist or trauma surgeon) to pre-hospital critically ill patients. This specialist medical care is supplementary to the existing regular ambulance care. The current study was performed by the HEMS of the Radboud University Medical Center Nijmegen (Lifeliner 3), which is stationed at the Military Air Base at Volkel, The Netherlands, with a deployment area close to 10.000 km^2^ and 4.5 million inhabitants. Based on pre-defined scramble criteria the HEMS is activated in a parallel fashion or could be activated at the request of the ambulance crew on scene [19].

## Results

### HEMS feasibility

50 patients were included in this study. Patient characteristics and reasons for HEMS deployment are shown in Table 1. In the trauma group 23 patients required intubation. Overall there were 22 traumatic brain injuries, 11 thoracic trauma patients and 12 multiple trauma patients. Nine patients in the emergency group had a reduced consciousness with loss of airway reflexes. Fourteen patients in the medical group were intubated.

**Table 1:** Patient characteristics

M/F	37/13
Age (yr)	50 [34–68.25]
Weight (kg)	85 [75–100]
Medical emergencies	16
Number of intubated patients	14
Intoxications	6
Neurological	4
Sepsis	4
Burns	1
Drowning	1
Trauma emergency	34
Number of intubated patients	23
Traffic accidents	15
Fall from height	12
Violence	3
Strangulation	2
Entrapment	1
Horse related	1
SpO_2_ (%)	96.5 [91.5–98]
HR (beats/min)	91 ± 34
SBP (mmHg)	140 ± 53
DBP (mmHg)	83 ± 34
MAP (mmHg)	102 ± 39
Revised Trauma Score	9 [8–12]
Glasgow Coma Score	7.5 [3–15]
Distance to emergency (km)	36 [24.75–44.50]
Flight time (min)	14 [11–19.25]
Prehospital time (min)	40 [31–52.5]

Data are expressed as numbers, mean ± SD or median [interquartile range] if not normally distributed according to D’Agostino & Pearson omnibus normality test

*HR* heart rate; *SBP* Systolic Blood Pressure. *DBP* Diastolic Blood Pressure; *MAP* Mean Arterial Blood pressure

All patients could be easily connected to the four skin patches required for ICON measurements. In the experience of the attending HEMS caregivers application of the device was easy and did not result in increased duration of on-scene time. Patch adhesion was reported as a concern due to clammy skin in 22% of all cases. Measurements were performed during medical care given on the scene of the accident and/or during transport by ambulance or helicopter. A total of 684 advanced cardiac output measurements during 896 min of registration were recorded, meaning that 24% of the potential measurements were not recorded by the device.

Measuring time and the number of recorded measurements per patient varied a lot between patients respectively (3 to 37 min) and (3 to 34 min). A total of 217 measurements were retrieved during medical emergencies and 467 measurements were retrieved from trauma patients. CO measurements ranged from 2.47 to 22.21 Lmin^−1^. Hemodynamic measurements of trauma and medical emergencies are registered in Table 2.

**Table 2:** Hemodynamic properties of trauma and medical emergencies

	Trauma emergencies	Medical emergencies	P
Heart rate (beatsmin^−1^)	85.3 [72–10.78]	96.4 [85.3–129]	0.05
Stroke volume (mL)	88.0 [72.7–135.2]	112.8 [86.8–133]	0.15
Cardiac output (Lmin^−1^)	8.2 [6.5–10.6]	10.8 [8.9–14.0]	0.02
SBP (mmHg)	147.6 ± 8.8	123.4 ± 14	0.13
DBP (mmHg)	89.4 ± 5.7	69.1 ± 8.0	0.05
MAP (mmHg)	109.5 ± 6.5	85.2 ± 9.5	0.04
SVR (dyne s cm^−5^)	1124.0 ± 166.0	638.4 ± 78.4	0.06

Data are expressed as mean ± SD or median [interquartile range] were appropriate

*HR* heart rate; *SV* stroke volume; *SBP* Systolic Blood Pressure; *CO* Cardiac Output; *DBP* Diastolic Blood Pressure; *MAP* Mean Arterial Blood Pressure. *SVR* Systemic Vascular Resistance. Mann Whitney U test or Unpaired T-test were used to test for statistical significance as appropriate, depending on the distribution of the data

**Fig. 1:**
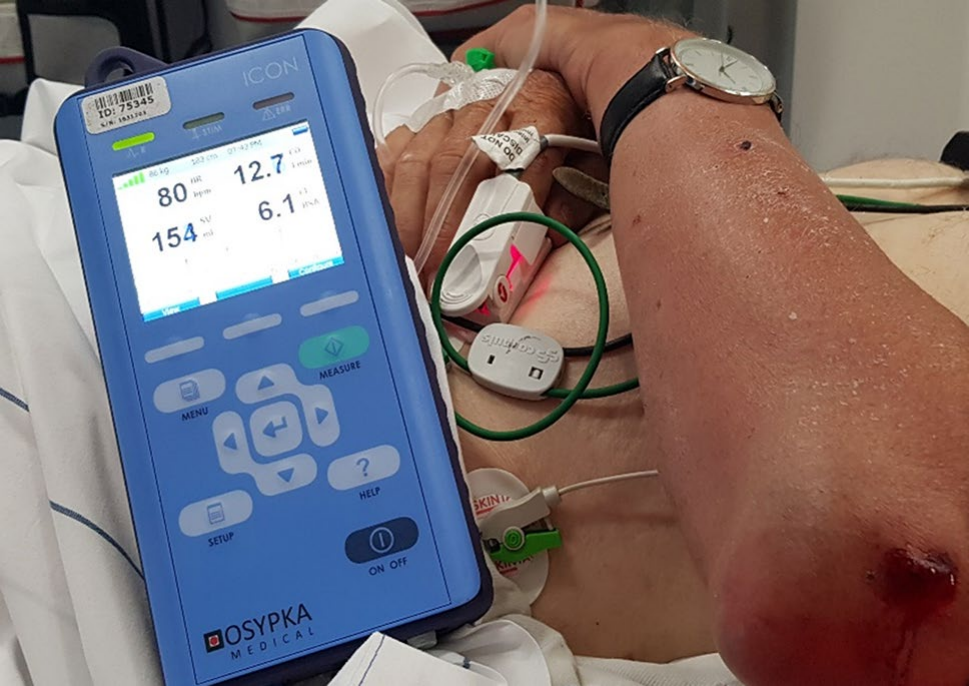
ICON connected to a patient. Size and weight of this device size, weight (20 cm × 10 cm × 4 cm; 1.36 kg). Display shows heart rate, cardiac output, cardiac index and stroke volume. SQI can been seen as green bars in the top left of the screen

Given its size, weight (20 cm × 10 cm × 4 cm; 1.36 kg; Fig. 1) and ease to clean, this device was considered practicable for working in this prehospital helicopter setting.

### Signal quality

**Fig. 2:**
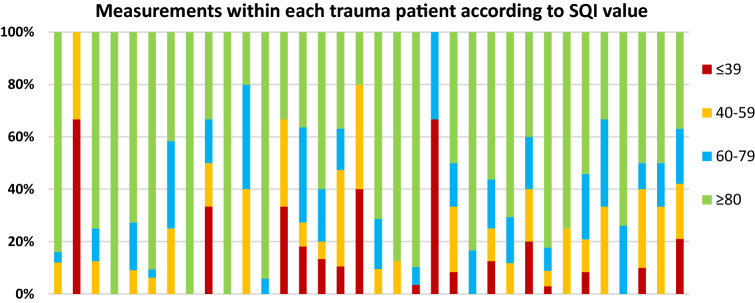
All measurements of each trauma patient are represented as bars and subdivided by SQI value. The total number of measurements within each patient is expressed as 100%. *SQI* signal quality indicator

**Fig. 3:**
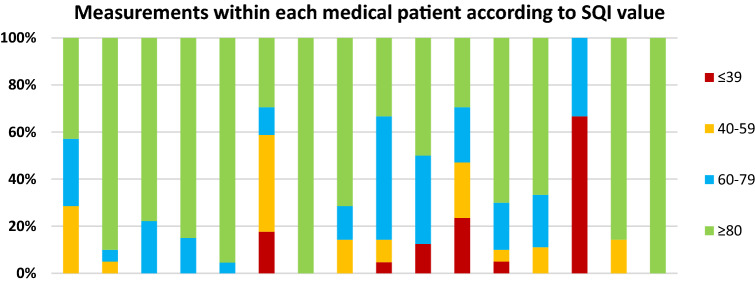
All measurements of each medical patient are represented by bars and subdivided by SQI value. The total number of measurements within each patient is expressed as 100%. *SQI* signal quality indicator

Of all recorded measurements 5.8% had an SQI < 40, 11.4% had an SQI of 40–59, 14.9% had a SQI between 60 and 79 and 67.8% had SQI ≥ 80. In 47 (94%) patients at least 1 measurement with a SQI ≥ 80 was recorded. In Figs. 2 and 3 the distribution of SQI values of the measurements within each trauma or medical patient are presented. A wide variation of the SQI value of the measurements was observed within a number of patient. In 3 patients (6%), one in the medical and two in the trauma emergency group we were unable to record any measurement with a SQI ≥ 80, which could not be attributed to too little registration time. Both trauma patients had severe thoracic trauma with accompanying pneumothoraces, which could explain this. A specific reason for the poor quality in the patient suffering from the medical emergency was not found, and could not be related to technical problems reported by the caregivers.

**Fig. 4:**
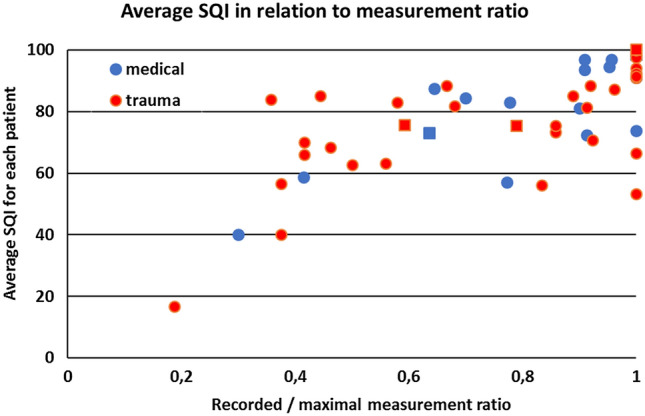
The ratio between number of recorded measurements and the number of maximal possible measurements per patient (given the patient’s measurement interval) is expressed against average SQI of all measurements in each individual patient. If the average SQI is very low, this seems to be accompanied by a low measurement ratio. Probably reflecting difficult measurement. Squares are patients transported through the air by helicopter. *SQI* Signal quality indicator. *Medical* medical emergency patient. *Trauma* trauma emergency patient

The average SQI of all measurements in each individual patient is expressed as a function of the ratio between the actual number of recorded measurements and measuring time in Fig. 4. The 3 patients in which we could not measure a SQI ≥ 80 are situated on the bottom left. Five patients had measurements taken during helicopter transport (represented as squares in Fig. 4). The quality of the data recorded in the helicopter was generally good indicating that transportation by helicopter does not interfere with the measurements. There was no chronological trend in the quality of data in any subject.

## Discussion

This is the first study to investigate the feasibility of electrical cardiometry for advanced hemodynamic monitoring measurements in the prehospital care provided by the HEMS. Patients could all be easily and quickly connected to the device without loss of on scene time. The ICON is a small and lightweight device, which is easy to operate and easy to clean. In 47 (94%) patients at least 1 measurement with a SQI ≥ 80 was recorded, the threshold above which the quality of measurements are regarded high enough to be used for clinical decision making [13]. In our study 68% of all measurements had SQI ≥ 80. Evaluation of user experience showed no insurmountable problems. Taken together this study shows that cardiac output monitoring using EC in prehospital helicopter care is feasible.

Emergency care in western countries has dramatically improved in the past decades. Both in the prehospital and in-hospital phase, quality of care has improved due to a number of reasons, among which are improvement of clinical decision rules based on big data, the availability of high-tech equipment (like ultrasonography), and the introduction of HEMS to enable specific care by a medical specialist on the prehospital scene. However, the basic treatment of the patient in shock basically has not changed. Shock is a mismatch of oxygen consumption and delivery leading to organ failure. Shock is often caused by a (relatively) low cardiac output state, and the cornerstone of the treatment of shock and the prevention of organ failure is to optimize cardiac output. In accordance, preventing organ failure before ICU admission improves outcome [20, 21]. However, as classical measurements of cardiac output (thermodilution or pulmonary artery catherization) are not suitable for the prehospital setting, new, small, reliable, easy to use, non-invasive methods for advanced hemodynamic monitoring are warranted. Shoemaker et al. found non-invasive measuring devices easier to use, quicker and cheaper than invasive monitoring [5, 22], and non-invasive measuring devices have been shown to help identifying patients at risk in the emergency department [23, 24]. The sooner these patients-at-risk are identified, the sooner potentially.

The EC signal is very sensitive to interference, leading to inaccurate measurements [12]. The changes in conductivity created by the circulatory system measured by the two inner electrodes are very small. Filtering techniques are needed to reduce noise to signal quality [11]. Patient factors (movement artefacts during treatment or transportation, movement of wires, clammy skin or electrode disconnection) or device factors (poor signal due to placement error) or both, all negatively influences the quality of the measurement [12]. In the prehospital HEMS operation all of the above are present and are jointly responsible for the 24% of missing recorded measurements as poor signal to noise ratio measurements are not stored on the ICON device.

Transport of a patient in an ambulance is a relative smooth ride compared to transportation by helicopter. The presence of vibrations theoretically could influence EC measurement. However, we acquired good quality measurements during helicopter transport. Due to the frequent sampling of the device, hemodynamic measurements could be recorded for all patients. Quality of the signal remained poor, indication some kind of interference with the registration. Caution should be taken to use these data for clinical decision making.

Two of our patients who did not have a good SQI, had an obvious pneumothorax (1 right and left, 1 right) requiring prehospital thoracic drainage. As air insulates, negative effects on the thoracic conductivity can be expected. Not only the type of injury, but also movement artefacts and the electrode position [25] or a combination of both can negatively affect the signal quality.

Measuring time varied a lot between patients. The most important reason of this large variation was caused by the clinical condition of the patient at the start of care. The best moment to connect the patient to the device was decided by the HEMS team, often after initial lifesaving care had been given. As transportation time was also variable, this also had an influence on measuring time. The number of recorded measurements per patient varied also between patients. This large variation is caused by the combination of varying recording duration and varying quality of the measurements.

Hemodynamic data of the medical emergencies in our study resemble the hemodynamic properties observed during periods of sepsis or inflammation [26]. The clinical problems in this group were mostly sepsis and intoxications. In the trauma emergency group we had 22 neurotrauma patients who were treated with vasopressors to increase blood pressure to maintain a cerebral perfusion pressure between 60 and 70 mmHg [27]. As expected, hemodynamic data in these patients are in correspondence with this treatment: a high blood pressure was observed in combination with a relatively low CO, reflecting the high systemic vascular resistance induced by vasopressors. This illustrates how EC measurements can aid in identifying the cause of shock, similar to its use in in-hospital emergency care [24]. However, as this is the first exploration of the use of CO as an additional vital parameter in pre-hospital care this needs further exploration.

This study is limited by the fact that this was a single centre study with a limited number of patients. Furthermore, EC measurements could have been influenced by arrhythmias, movement artefacts and incorrect electrode placement [25]. We have not evaluated these factors, but rather assumed that such artefact would result in a low SQI. However, technically it is possible to find SQI ≥ 80 despite the fact that electrodes are not correctly placed. All 14 doctors and 9 nurses were trained to familiarize them with the ICON and to optimize its use including correct electrode placement. Including this training we think we have minimized the risk of electrode malposition.

Most patients were treated for their injury or pathophysiology before the first measurements were performed. Because of this we have limited measurements during profound hypovolemic shock. This could be seen as a limitation of our study.

In conclusion, advanced hemodynamic monitoring using EC during prehospital care provided by the HEMS is feasible. EC may therefore be a promising candidate to aid in prehospital clinical decision making in critically ill patients.

## Data Availability

The datasets during and/or analyzed during the current study are available from the corresponding author on reasonable request.
